# Chinese EFL University Students’ Self-Efficacy for Online Self-Regulated Learning: Dynamic Features and Influencing Factors

**DOI:** 10.3389/fpsyg.2022.912970

**Published:** 2022-07-06

**Authors:** Qi Xu, Jin Wu, Hongying Peng

**Affiliations:** ^1^Center for Linguistics and Applied Linguistics, Guangdong University of Foreign Studies, Guangzhou, China; ^2^School of English for International Business, Guangdong University of Foreign Studies, Guangzhou, China

**Keywords:** self-efficacy for self-regulated learning (SESRL), online learning environment, dynamic features, influencing factors on SESRL, online language pedagogy

## Abstract

Self-efficacy is crucial for successful self-regulated learning, particularly in an online environment, yet research on self-efficacy for online self-regulated learning has received relatively little empirical attention in the language education domain. In this study, we investigated the dynamic features of English as a Foreign Language (EFL) university students’ self-efficacy for self-regulated learning (SESRL) in the online environment, and explored the influencing factors on SESRL. Multiple sources of data (questionnaires, reflective journals, and semi-structured interviews) over a period of one semester were collected, analysed, and triangulated. Our results demonstrated that most students displayed a relatively constant and high self-efficacy for self-regulated online learning, and that a few students also experienced an increased or decreased SESRL. Thematic analysis further revealed a variety of task-, learner-, course-, and technology-level factors contributing to learners’ self-efficacy beliefs for self-regulated online learning. Our findings thus offer pedagogical implications for self-regulated foreign language learning in an online context.

## Introduction

Online education has witnessed a tremendous boom in the field of second language (L2) learning. While students are given more flexibility and convenience in an online environment to decide the content and the pace of their L2 learning, they also need to take more responsibility in planning and managing their learning behavior (Artino and McCoach, 2008). In other words, self-regulated learning (SRL) is of particular importance for students’ online language learning.

Self-regulated learning is an active and constructive process whereby students set goals for their learning, and monitor, regulate and control their cognition, motivation, and behavior based on the goals they set and contingent upon the contextual features of the environment (Pintrich, 2000). Despite the importance of SRL in the online context, most SRL studies were conducted in traditional classrooms and much research attention has been placed on the identification, evaluation and cultivation of self-regulated learning strategies (e.g., Zimmerman and Moylan, 2009; Bai and Wang, 2020; Teng and Zhang, 2020). It should be noted that self-regulation strategies alone do not necessarily lead to students’ effective SRL (Creer, 2000).

Another necessary component for the SRL process is students’ high self-efficacy for self-regulated learning (SESRL), namely students’ beliefs they hold in applying self-regulated strategies (Usher and Pajares, 2008). Previous studies have found that self-efficacy is significant for students’ successful application of SRL strategies such as knowledge rehearsal, goal-oriented monitoring, idea planning, peer learning, and interest enhancement (Teng, 2021), and can predict students’ language achievement in the end (Mills et al., 2007). SESRL can also mediate the relationship between task behaviors and academic performance (Alghamdi et al., 2020). Previous research in this regard often collected questionnaire data at one time point. However, as Dörnyei (2000) rightly pointed out, students’ self-belief is not static and fixed; instead, it is an ongoing, dynamic, and discursively constructed process. In light of this, it would be difficult to envisage the trajectory of SESRL through those cross-sectional questionnaire data. It is thus warranted to explore English as a Foreign Language (EFL) learners’ SESRL in the online environment over a longer period of time (e.g., over one semester), which would be the focus of the present study.

Another goal of the study is to further explore relevant and important factors influencing EFL learners’ online SESRL, with a view to establishing conditions helpful for learners’ SE enhancement and their successful online learning achievement. Although scholars have identified various factors influential for learners’ self-efficacy (SE; Shen et al., 2013; Broadbent and Poon, 2015), they paid little attention to the specific learning context, a variable also essential for L2 learning and especially highlighted in recent language learning research (e.g., Sato and Storch, 2020). As such, it is necessary and important to explore how task characteristics combined with contextual features are related to language learners’ SE beliefs and their online SLR processes.

## Literature Review

### Self-Regulated L2 Learning in the Online Environment

Self-regulated learning is usually regarded as an active and constructive process, whereby students set goals for themselves, monitor, regulate and control their cognition, motivation and behavior on the basis of the goals they set and the contextual features of the learning environment (Pintrich, 2000; Zhang and Zhang, 2019). SRL has been extensively investigated in L2 education in recent years (Dent and Koenka, 2016). It has been shown that SRL is effective in enhancing different language skills and specific language features, such as writing (Sun and Wang, 2020), reading (Mohammadi et al., 2020), listening (Tan et al., 2019), and vocabulary (Choi et al., 2018). Studies like Bai and Wang (2020) have also shown that, when equipped with SRL strategies, students tend to show positive attitudes toward language learning, engage in L2 learning activities beyond classrooms and class hours, and in the end become active, lifelong learners. Teng and Zhang (2022) thus proposed that “a self-regulation approach would be especially conducive to promoting active and productive learning of a second/foreign language (p. 588).”

The identification and cultivation of SRL strategies have become an increasingly prominent issue in an online environment, because, different from traditional face-to-face classroom settings where language teachers exercise control over students’ learning processes and monitor student attention and progress, online settings have different affordances for language learning. Specifically, online learning is more flexible and adaptive, providing learners access to authentic, contextualized, and enhanced learning materials with which they can involve themselves in collaborative learning tasks and activities. Accordingly, L2 students need to be equipped with effective SRL strategies and become more self-regulated in their learning in online settings (Artino and McCoach, 2008). In a meta-analysis of SRL strategies in online settings, Broadbent and Poon (2015) found that SRL strategies such as time management, metacognition, critical thinking and effort regulation had significantly positive correlations with students’ academic success, while strategies such as rehearsal, organization and elaboration were found to be least empirically supported in online environments. Researchers (e.g., Godwin-Jones, 2011) have also delved into factors that influence L2 learners’ employment of SRL strategies in online environments. For example, Godwin-Jones (2011) found that blogs, online editors, e-portfolios, e-logs and online language labs are helpful for improving L2 learners’ SRL strategies.

However, knowledge of self-regulation strategies alone does not necessarily lead to students’ effective SRL (Creer, 2000). Another prominent factor that determines students’ engagement in the self-regulation process is the beliefs they hold about their capabilities to regulate their learning, i.e., SESRL (Usher and Pajares, 2008). It is increasingly evident in literature that students’ self-efficacy beliefs influence the goals they set for themselves, the course of action they choose to pursue, the challenges as well as their learning behaviors along the way, and the outcomes they expect their efforts to produce (Bandura, 2006).

### Self-Efficacy for Self-Regulated L2 Learning

Self-efficacy for self-regulated learning refers to the beliefs that students hold about their ability to use SRL strategies to enhance their learning, acquire academic skills, and reach their goals (Zimmerman and Cleary, 2006). These beliefs are particularly important in the academic domain with language learning included, because they are significantly related to students’ academic outcomes, such as grades, classroom engagement, homework practices, as well as students’ actual self-regulation skills (e.g., Zimmerman and Kitsantas, 2005; Caprara et al., 2008).

In spite of its importance, SESRL is still an underexplored construct in L2 research. Much research attention has been devoted to exploring self-efficacy in a general sense (Yun et al., 2018) or self-efficacy in particular, such as L2 self-efficacy belief for language skills (e.g., writing, speaking, reading, listening), (e.g., Zare and Mobarakeh, 2011; Yan, 2012; Ho, 2016; Leeming, 2017). Recently, language learning researchers are increasingly exploring the role of SESRL in influencing learners’ application of a variety of SRL strategies such as knowledge rehearsal, goal-oriented monitoring, idea planning, peer learning, and interest enhancement (Teng, 2021), and in facilitating language achievement (Mills et al., 2007).

Additionally, an emerging research trend worthy of noting is that learners’ self-efficacy beliefs are often not regarded as static and fixed, but as fluctuating and dynamic in nature (Dörnyei, 2000). In light of this view, there are empirical studies on self-efficacy conducted from a longitudinal perspective. For example, Phan (2013) investigated the growth in academic self-efficacy among 196 middle school students over 2 years, and found that students’ self-efficacy increased between the first and third time points (with a seven-month time span) but decreased at the fourth time point. Also adopting a longitudinal approach, Piniel and Csizér (2014) investigated changes in self-efficacy, motivation and anxiety among university students studying academic English writing. Specifically, with a mixed method research design, Piniel and Csizér (2014) collected data from questionnaire, academic writing test, and students’ essays six times over the course of a 14-week semester. Their results disclosed a discrepancy between a linear decrease in self-efficacy reflected in the quantitative data and an improved self-efficacy reported in qualitative data. Leeming (2017) collected data from questionnaires and interviews over 1 year and found growth in SE over the course of the study. In general, longitudinal studies on SE used panel data to examine the changes of students’ self-efficacy beliefs over time, with qualitative data like students’ essays or interviews being used to provide insights and explanations for the changes revealed by quantitative data. To date, however, little empirical evidence has been yielded regarding how SESRL would change over a longer period of time and what factors potentially contribute to the changes in SESRL. The present study thus takes an initiative in doing so, results of which may shed important light on the dynamic, discursively constructed feature of SESRL in the online environment.

### Self-Efficacy for Self-Regulated Learning in Online Environments

SESRL plays an important role in online learning environments, as students are expected to be responsible for their learning process compared to those in traditional face-to-face learning settings. Students who believe they can use their self-regulatory skills to help them learn are more motivated in engaging in the learning process and more persistent in accomplishing difficult learning tasks, which in turn render the students more competent in their online learning (Alghamdi et al., 2020). Joo et al. (2000) also found that students’ SESRL was positively related to their strategy use in web-based learning. Considering its particular importance in online settings, it is of great value to explore the factors that may contribute to, or hinder the development of SESRL.

Previous research has divided factors potentially influencing SE into different categories (Bandura, 1997): mastery experience, vicarious experience, social persuasion, and physiological state. Specifically, mastery experience is considered the most important source of SE information (Usher and Pajares, 2008). Positive and successful past experiences enhance one’s SE abundantly (Britner and Pajares, 2006), while negative and unsuccessful experiences would dampen one’s SE (Pajares, 2003). SE can also be developed by observing others performing a task successfully (i.e., via vicarious experience); that is, learners persuade themselves that if others can do it, they can do it better (Bandura, 1997). The third source is social persuasion. Verbal and non-verbal feedback from others influences the development of SE. For example, positive feedback from teachers may enhance students’ SE (Zeldin and Pajares, 2000). Physiological state, the fourth source of information, relates to a person’s psychological or emotional state. For example, negative feelings such as anxiety may lower one’s SE (Usher and Pajares, 2008).

Empirical studies exploring influential factors of SE in online settings yielded findings partly supportive for Bandura’s (1997) classification. For example, it has been found that mastery experience such as computer experience (Jashapara and Tai, 2006) and previous success with online learning technology (Bates and Khasawneh, 2007) were significantly related to self-efficacy for online learning. Positive feedback from peers (Chu and Chu, 2010) and learner-learner interaction (Lim et al., 2016) were also found to be in close relation with students’ computer self-efficacy. However, online learning environments may provide different sources for SE development. For example, Lin et al. (2013) examined sources underlying middle-aged and older adults’ internet SE and found that—in addition to the four sources mentioned in Bandura (1997)—proactive personality was another important source of SE development. More recently, Alonso-Mencía et al. (2021) found that students’ gender and nationality were also closely related to their SE for learning, as well as their performance in massive open online courses (MOOCs).

The aforementioned studies exploring the factors that affect SE mainly focused on computer SE, internet SE, or online learning SE. To the best of our knowledge, however, little is known about the factors influencing SE for online SRL. This is an important issue, perhaps especially in today’s technologized world “where L2 learners have access to diverse and myriad learning resources that articulate with their personal goals, learning interests and preferences, prior knowledge and language and digital competencies” (Peng et al., 2020, p. 87). The only study that investigated this issue is Alghamdi et al. (2020), who found that learner-related (e.g., gender) and task-related (e.g., task type) factors may exert distinct influences on SESRL in online settings, as compared to traditional classrooms. More empirical studies are thus needed to systematically explore factors (e.g., learner-related, task-related, context-related) that exert an influence on students’ SESRL.

### Research Questions

We propose that SESRL in online learning environments is an important but neglected issue. Little is known about how the construct develops over time and the factors that influence the construct have rarely been systematically studied. In the present study, we adopted a mixed-method design, examined the dynamic features of EFL learners’ SESRL in online environments and their evolution over a period of one semester, and explored possible factors that influence learners’ SESRL. We formulated the following research questions:

Research Question 1: What are the dynamic features observed in terms of EFL students’ self-efficacy for online self-regulated learning over a period of one semester?

Research Question 2: What are the factors influencing EFL students’ self-efficacy for online self-regulated learning?

## Methods

### Participants

The participants in our study included 52 sophomores (42 females and 10 males) majoring in International Business at a Chinese university. Their ages ranged from 19 to 21, with a mean age of 19.8 (SD = 0.61). They were intermediate-level EFL students with an average International English Learning Testing System (IELTS) mock test score of 6 (SD = 4.42), and an average English learning experience of 12 years. Over the period of a 16-week semester, the students took a comprehensive business English course intended both to develop their language skills and construct theme-based business knowledge. Each week, the students met online for two sessions, with each lasting 80 min (session 1: online self-regulated learning; session 2: online live class). The instructor of the course held a Ph.D degree in applied linguistics and had over 6 years’ experience in teaching business-English-related courses.

### Online Learning Context

The e-learning platform MosoTeach was selected for students’ SLR in the first session of the class. MosoTeach is a popular e-learning management platform developed in China, and its two versions—a mobile application and an online website—enable teachers and students to use their smartphones or personal computers to log into the system. Students can view the learning resources in multiple formats (e.g., videos, files, pictures, and recordings), take online quizzes, submit completed assignments, receive teacher feedback, and give peer feedback. A pre-study questionnaire showed that 85% of the students had used MosoTeach prior to our study, and approximately 50% were familiar with its functions.

In the current study, MosoTeach was used as the platform for students’ online SRL, since it can give students both the freedom to manage their own learning pace and the opportunity to improve their SRL abilities.

### Online Self-Regulating Learning Procedures

**FIGURE 1:**
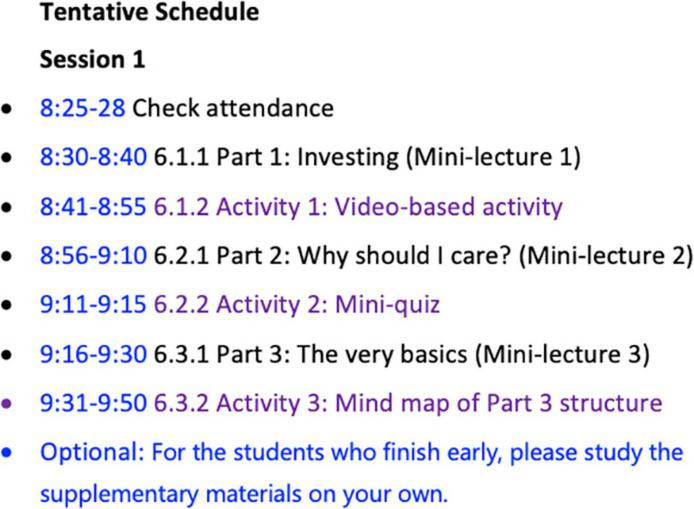
An example of a tentative schedule of an SRL session.

During the SRL session, the students were required to participate in the online activities following the class schedule (Figure 1). It should be noted that the timetable was only for reference, and the students could adjust their own learning speed as long as they could finish all the tasks within the allocated 80 min.

**FIGURE 2:**
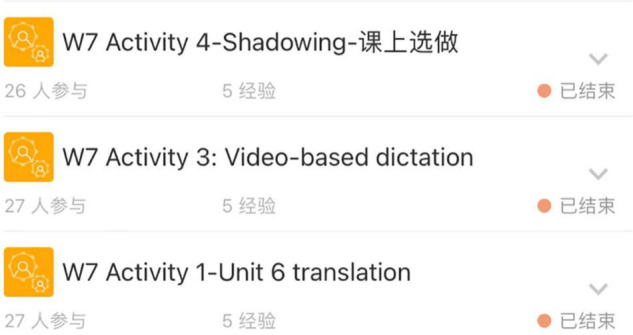
An example of MosoTeach activities (week 7).

**FIGURE 3:**
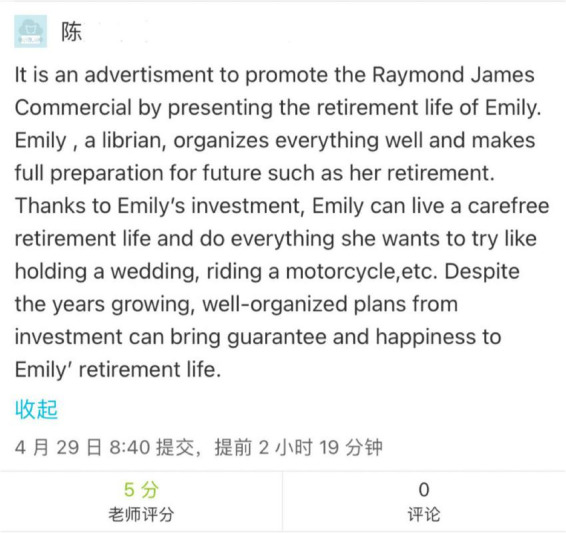
An example of students’ completed work (week 6).

The general procedures involved the following. The students first watched the mini-lectures (each lasting 5–10 min) created and recorded by the instructor, which covered a wide range of topics on a specific theme from the textbook content (including leadership, investment, money and banking, and business ethics). Figure 2 lists the learning materials for the introduction of Investing in Week 6, including three recorded mini-lectures targeting different aspects of investment. The students were then asked to complete a series of tasks based on the recorded mini-lectures, such as questions and answers, brainstorming, translating, summary writing, quizzes, and mind map drawing of an article structure, and upload their completed work to MosoTeach to be scored by the instructor at a later time (Figure 3).

### Research Instruments

Using a mixed-methods approach, quantitative and qualitative data were collected via questionnaires, reflective journals, and semi-structured interviews.

#### Questionnaires

Two questionnaires were designed and distributed to the students throughout the semester. Questionnaire 1, distributed in Week 1, was used to obtain students’ background information, such as their English learning experiences and general attitudes toward the course. Questionnaire 2, distributed in in Weeks 3, 9, and 16, respectively, contained 6-point Likert scale questions to compare students’ self-efficacy throughout the semester and to uncover factors influencing self-efficacy.

Questionnaire 2 consisted of different parts. One part included six items, adapted from Artino and McCoach (2008), which was used to collected information on students’ self-efficacy for online SRL. Specifically, the six items gauged three different constructs (i.e., students’ ability to deal with difficulties, confidence in self-regulation, and confidence in task performance). The construct validity, criterion-related validity, and internal consistency reliability of these items have been reported of adequacy in Artino and McCoach (2008).

In addition, the questionnaire also contained several scales collecting information that potentially reveals important factors influential for students’ SE for online SRL, as listed below with Cronbach Alpha reliability indexes calculated and reported (α = 0.891).

• Five items for students’ attitudes toward the online course, which were also adapted from Artino and McCoach’s (2008) survey. Together with self-efficacy, this scale has been proved to be a critical predictor of students’ SRL competence in online contexts;
• Four items for students’ attitudes toward online tasks, including their willingness to participate in the tasks and their perceived usefulness of the tasks (with reference to Kormos and Dörnyei, 2004);
• Six items for students’ acceptance of MosoTeach for online SRL, in which the items were extracted from the technology acceptance model questionnaire (Huang et al., 2012) and modified to address the characteristics of MosoTeach. This scale included three constructs: perceived ease of use, perceived usefulness, and behavioral intention;
• open-ended questions concerning students’ critical comments on and suggestions for online SRL, in which the students were asked to write their opinions about online SRL, including general attitudes, online learning materials, online activities, and assessment methods.

#### Reflective Journals

Students were asked to write reflective journals about their online SRL experience in Weeks 2, 3, 6, 10, and 16. These journals were used to explore the factors that may influence students’ SESRL in an online learning context. Some writing guidelines were provided, including general attitudes toward the online course, improvements they had made in English learning via online SRL, difficulties they had encountered in the process, and how they solved the problems. They could write in either Chinese or English. Chinese reflective journals were translated into English by a research assistant and double-checked by the researchers for the purpose of this research.

#### Semi-Structured Interviews

To further explore factors affecting students’ SESRL in online environments, by means of extreme case sampling (Dörnyei, 2007), 19 students were selected for semi-structured interviews with the researcher, based on their scores of SESRL in the questionnaire. The full score for the SESRL scale with six items was 36 points (6 × 6). Five students with high SE (an average score of 34.33), five with low SE (an average score of 22.50), four with increasing SE (an average increase of 6.50 points), and five with decreasing SE (an average decrease of 5.50 points) were chosen. The interviewees were asked questions such as “What is your overall impression of online SRL via MosoTeach?” “How do you evaluate your ability in online SRL, including strengths and weaknesses?” “Are there any changes of your ability in online SRL throughout the semester? If yes, what are the possible reasons for such changes?” Follow-up questions were also posed based on the interviewees’ response. Each interview lasted 10–15 min and was conducted in Chinese. The recordings of all interviews were then transcribed and translated into English by a research assistant who majored in English applied linguistics. The translation was double-checked by the researchers to verify its accuracy.

### Data Analysis

To answer the first research question concerning the dynamic features of students’ SESRL in online environments, the scores of the questionnaire items concerning SE were calculated and compared among Weeks 3, 9, and 16 to examine whether significant changes occurred.

In response to the second research question (factors that have influenced students’ SE for online SRL), data from Questionnaire 2, reflective journals, and semi-structured interviews were analysed and triangulated. To identify and analyse the influential factors that emerged, we adopted the thematic analysis method proposed by Braun and Clarke (2006), which allows researchers to summarize, highlight key features, and interpret a wide range of data sets. Specifically, our analysis followed a 6-phase process (Braun and Clarke, 2006) which included: transcribing the data, reading the data, systematically coding interesting features emergent from the data, searching for themes (factors) by collating and grouping the emergent codes together to create overarching themes, reviewing the themes (factors) identified, and producing the report. Coding and analysis of the qualitative data were made by the first two authors, who reached 90% agreement on the analysis. Discussions were then made on controversial items until consensus was achieved.

## Results

### Students’ Self-Efficacy for Self-Regulated Learning in Online Environments

**FIGURE 4:**
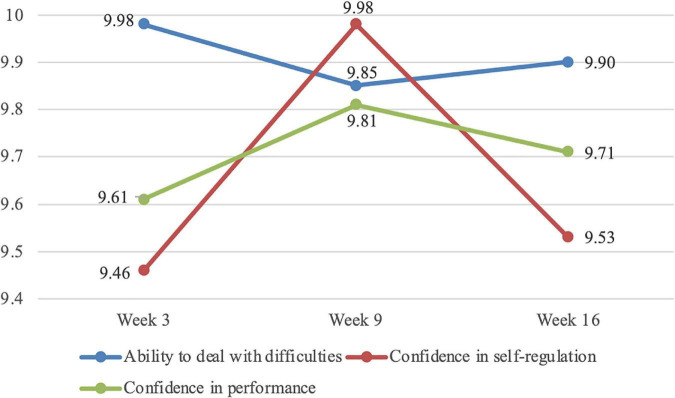
Changes of students’ SE for online SRL throughout the semester.

To examine the dynamic features of students’ SESRL in online environments throughout the semester, data collected with Questionnaire 2 in Weeks 3, 9, and 16 were collated, compared, and analysed (Figure 4).

Initially, the results show that the majority of students held relatively high SESRL in online environments (all scores above 4.5 on a scale of 6), especially for the construct of “perceived ability to deal with difficulties.” Specifically, albeit with technical obstacles and difficult materials, students were mostly confident that they were able to learn and understand the learning materials on their own throughout the semester. Slight changes were found in the construct of “confidence in task performance,” with the mean score increasing from 9.61 (SD = 0.634) in Week 3 to 9.81 (SD = 0.812) in Week 9, and dropping to 9.71 (SD = 0.756) in Week 16. Regarding the construct of “confidence in self-regulation,” obvious changes were observed with a sudden increase from 9.46 (SD = 0.685) in Week 3 to 9.98 (SD = 0.832) in Week 9, and then a decline to 9.53 (SD = 0.793) in Week 16. The results indicate that, in the middle of the semester, students gained more confidence in their ability of self-regulating learning even without the presence of the teacher, and higher evaluation of their capability to perform well in English learning activities.

**FIGURE 5:**
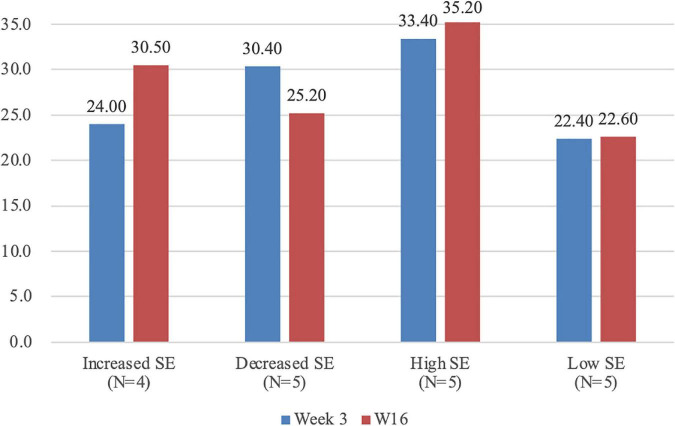
Individual differences in students’ SE for online SRL.

Although no statistically significant changes of SESRL were detected from Week 3 to Week 16 (*F*[2, 153] = 0.517, *p* = 0.60, η^2^ = 0.25), a closer look reveals individual differences in students’ self-evaluation of their capabilities in online SRL throughout the semester (Figure 5). A comparison of students’ SE between Week 3 and Week 16 showed that scores of four students’ SE increased by 6.50 points on average, and those of five students decreased by 5.20 points on average. Additionally, five students’ SE remained high with an average total score of 34.33, while five students held low SE with an average score of 22.50. The scores of the rest remained relatively stable.

Many factors could have influenced the differences in students’ SE for online SRL. The following section presents the major findings.

### Factors Influencing Students’ Self-Efficacy for Self-Regulated Learning in Online Environments

To determine the factors that may have influenced students’ levels of SESRL, the reflective journals of all students and transcribed interviews of the 19 students were subjected to thematic analysis, which revealed several interdependent macro-themes/factors, including task-, learner-, course-, and technology-level factors (see Table 1). The results concerning task-level factors were found to be the most critical in affecting the students’ SESRL, and are thus addressed first.

**TABLE 1:** Coding scheme of factors influencing students’ SESRL in online environments.

Different levels	Factors (positive)	Factors (negative)
Learner-level factors	Self-discipline	Lack of self-discipline
	Self-adaptation	Lack of self-adaptation
Course-level factors	Positive course attitudes	Negative course attitudes
	Multitudinous resources	Limited resources
Task-level factors	Diversified task design	Repetitive task design
	Appropriate task load	Heavy task load
Technology-level factors	Technical knowledge	Technical obstacles

#### Task-Level Factors

Before discussing task-level factors, it is necessary to examine the questionnaire results in terms of students’ attitudes toward online SRL tasks, which mainly included watching recorded videos of course content and completing corresponding tasks on MosoTeach.

Table 2 shows that students held a positive attitude toward the tasks assigned in the online SRL process. However, toward the end of the semester, they became significantly less positive about watching the recorded videos or participating in MosoTeach activities (*t* = -2.22, *p* = 0.03; *t* = -2.02, *p* = 0.05). The decreased motivation to a certain extent may have affected their SESRL. The influencing factors have been further disclosed by findings from reflective journals and interviews as follows.

**TABLE 2:** Students’ attitudes toward online SRL tasks.

Attitudes toward online tasks	W3		W16		*t*	*p*
		
	Mean	SD	Mean	SD		
(1) I am willing to watch the recorded videos	5.75	0.523	5.46	0.727	–2.223	0.031*
(2) I think watching the recorded videos is useful for my learning	5.52	0.703	5.37	0.864	–0.969	0.337
(3) I am willing to participate in activities on MosoTeach	5.52	0.612	5.19	0.991	–2.020	0.049*
(4) I think the tasks on MosoTeach are useful for my learning	5.47	0.702	5.33	0.785	–0.895	0.375

*p < 0.05

##### Task Design

Some students mentioned the influence of tasks on changes in their SE. With students’ increasing familiarity with online task design, their interest in English learning activities on MosoTeach also enhanced from the start to the middle of the course. However, students also complained about the task design toward the end of the semester. For instance, Student 16 with a decreased SE said in the interview:

As learning progressed, online tasks became increasingly difficult. At the later stage of the online course, some topics were difficult to understand, and more writing tasks were assigned, which put me under a lot of pressure.

This could explain why students’ confidence in their self-regulated abilities and task performance decreased toward the end of the semester. One student pointed out that the weekly online tasks were similar throughout the semester—when she became more familiar with the class schedule, she became less focused. This could be another reason for the decreased SE of some students at the end of the semester.

##### Task Load

Five students in the interviews and eight students in their reflective journals complained about the heavy workload in the process of SRL, which made them anxious and hindered their ability to finish the tasks efficiently. For instance, Student 3 with a low SE stated in the reflective journal:

Sometimes the workload was too heavy in the fixed time of online self-regulated learning, and I would feel very nervous when doing the tasks on MosoTeach. That made me less confident in the learning process.

#### Learner-Level Factors

##### Self-Discipline

Students’ ability to discipline themselves to concentrate on the online learning process was first identified as a noticeable factor that positively or negatively affected their SESRL. When faced with distractions, 73.1% of the students were confident of learning the contents of the online course by the end of the semester, and 26.9% were less certain about their ability to manage and control their online SRL with distractions such as instant messages, entertainment facilities, and noise at home. The interview data showed that nine students with high or increased SE claimed they could concentrate on the learning materials with strong self-discipline, while the five students with low SE said they lacked the ability to focus and were easily distracted during the online learning process. Examples from student interviews follow:

This type of self-regulated learning accompanied by well-designed tasks helped improve my self-discipline. I was therefore capable of focusing my attention on this online course. (S12, high SE)

Because I have weak self-control and self-discipline, I always procrastinate especially when there is not a fixed deadline. (S5, low SE)

##### Self-Adaptation

Self-adaptation to the online SRL mode was identified as another factor. Four students with increased self-efficacy recalled that after one-semester of online SRL experience, they became familiar with the online platform and learning style, which increased their confidence in learning English via online SRL. Below is an example of one student’s opinion:

Compared with the beginning of the semester, I’m able to participate in the online learning activities without difficulty, because I’ve become more accustomed to online self-regulated learning. (S6, increased SE)

#### Course-Level Factors

##### Attitudes Toward the Course

The first course-level factor related to students’ SE was students’ attitudes toward the course. The questionnaire results (Table 3) show that throughout the semester, students had a positive attitude toward the online course, realizing its importance and effectiveness in enhancing their English learning. They considered it extremely important to learn from the materials and perform well in the online course. The usefulness of the course was rated with an increasing value toward the end of the semester (items 2 and 5). Therefore, the high perception of the online course may have contributed to their relatively high SE for online SRL.

**TABLE 3:** Mean scores of students’ attitudes toward the course.

Attitudes toward the course	W3		W9		W16	
			
	Mean	SD	Mean	SD	Mean	SD
(1) It is personally important for me to perform well in the course	5.50	0.577	5.58	0.637	5.63	0.595
(2) This course can provide a great deal of useful information	5.44	0.639	5.54	0.541	5.62	0.631
(3) It is important for me to learn the materials in the course	5.46	0.637	5.54	0.653	5.44	0.689
(4) I’m very interested in the content of the course	5.29	0.576	5.25	0.564	5.27	0.698
(5) The knowledge I gain from the course can be applied in many different situations	4.94	0.938	5.13	0.715	5.17	0.734

Interview data further substantiated the importance of course attitudes. Students who considered the online course important were more likely to take online learning activities seriously and monitor their learning behavior in a self-disciplined manner. However, four students with decreased SE admitted that they showed lower concentration toward the end of the semester because they were less motivated to learn, which negatively influenced their confidence in participating in online SRL. Examples of students’ statements follow:

I attach great importance to this course since I know clearly that my English proficiency is relatively low. In the online self-regulated learning process, I tried my best to keep up with the learning pace and improve myself. (S10, high SE)

In the last few weeks, I was looking forward to the summer holiday, so I didn’t take the course that seriously. I became less focused on the course toward the end of the semester. (S16, low SE)

##### Learning Resources

As indicated in the interviews and students’ reflective journals, the diversified learning resources increased students’ motivation for online SRL and enhanced SE. Access to various learning resources, such as business-related English videos, English business journal articles, or the latest business news, was considered a great advantage of the online course, because it afforded students precious opportunities to enhance their English language skills through self-regulation. For instance, one student stated:

The teacher provided us with a variety of English learning materials. I also searched for more learning materials on websites and studied by myself. This increased our interest in online learning of English and enhanced my ability of self-learning. (S13, high SE)

#### Technology-Level Factors

Technology acceptance of online learning platforms, such as MosoTeach, was found to be a critical influencing factor. The questionnaire results (see Table 4) show that 84.6% of the students strongly agreed that using these online platforms provided a positive English learning environment, 78.9% considered it easy and convenient for the learning activities, and 63.4% liked the online platforms to learn the Business English course. Confidence in using technology was critical for a smooth undertaking of the online course, while technology breakdowns may cause unsatisfactory learning experiences. Therefore, high technology acceptance is another factor that contributed to students’ relatively high SE.

**TABLE 4:** Average scores of students’ technology acceptance.

Attitudes toward online learning platforms	W3		W9		W16	
			
	Mean	SD	Mean	SD	Mean	SD
(1) Using these online platforms provided a positive English learning environment	5.12	0.899	5.27	0.745	5.25	0.813
(2) The learning materials cater for my study needs	5.17	0.785	5.38	0.661	5.25	0.764
(3) Using the online platforms for the learning activities was easy and convenient	4.94	0.895	5.10	0.869	5.02	0.980
(4) Learning through the online platforms enhanced my desire to use English	4.85	0.849	4.77	0.899	4.85	1.073
(5) I like using the online platforms to learn Comprehensive Business English	4.9	0.934	4.77	0.942	4.94	1.037
(6) If I have access to these online platforms, I will continue to use them to improve my English learning	4.92	0.926	5.02	0.828	4.88	1.078

Among the six questionnaire items regarding SE for online SRL, dealing with technical difficulties was not a problem for most students, with 84.6% of the students confident of their learning from online materials even when technical difficulties arose. Although the mean score dropped from 5.40 in Week 3 to 5.25 in Week 16, it was still the highest among all the SE items. Six students mentioned in their reflective journals that as the course progressed, they had learned to tackle the technological problems on their own or with the help of the teacher or their peers.

However, the interview data also revealed that a few students who were not able to handle network problems effectively felt frustrated by online SRL, especially at the beginning of the semester. These technical problems, if not resolved, could lead to negative attitudes toward online SRL, and thus low SE.

## Discussion

Using a mixed-method design, in this study we investigated the dynamic features of Chinese EFL students’ SE for online SRL in a business English course and uncovered various types of factors that influenced students’ SESRL. Specifically, quantitative analysis of the questionnaire data showed that most students held strong SE beliefs for online SRL and slight changes were observed in their SESRL over the course of the study (i.e., one 16-week semester). The questionnaire data together with qualitative analysis performed on the reflective journal and interview data revealed task-, learner-, course-, and technology-level factors that influenced students’ SE for online SRL. While some of the factors positively influenced students’ SESRL, others had a detrimental effect.

### Dynamic Features of Students’ Self-Efficacy for Self-Regulated Learning in an Online Context

In response to RQ1, the changes of students’ SE for online SRL were tracked throughout the semester. In contrast to most previous studies—in which only one-time SESRL was recorded (e.g., Joo et al., 2000; Alghamdi et al., 2020)—the present study revealed the relatively dynamic nature of students’ SESRL in an online learning environment. Although most students showed great confidence in the online SRL process, we still observed changes in their SESRL—namely students’ SESRL moderately increased in the middle of the semester and then dropped again at the end of the study. This dynamic feature of SESRL supports the view that SE is malleable over time (Young Kyo, 2022). However, in contrast to the researchers who reported that SE increased over longer period of time (i.e., more than 1 year; Phan, 2012; Young Kyo, 2022), we found that SESRL decreased slightly at the end of a shorter period of time (16 weeks). Similarly, Piniel and Csizér (2014) also found that students’ SE decreased over a period of 14 weeks.

The different patterns of SE may be attributed to the varying settings in which the studies were conducted. In the settings where factors such as realistic learning tasks, appropriate feedback (Woodrow, 2006), or classrooms focusing on students’ SE (Young Kyo, 2022) exist, students’ SE may improve. Conversely, factors such as the increased difficulty of the tasks (Piniel and Csizér, 2014) or the increasing complexity of academic demands (Caprara et al., 2008) may inhibit students’ SE development. In our study, we also found that task-related factors greatly influenced students’ SESRL.

In addition, individual differences were evident in students’ SE beliefs, as some students showed consistently high or low levels of SE while others displayed an increase or decrease in SE over time. That is, the students had distinctively different developmental trajectories in their SE, so combining them would produce a poorly defined one-size-fits-all conclusion. We thus propose to extend previous studies—which mainly focused on students’ SE at an aggregated level (e.g., Caprara et al., 2008; Shen et al., 2013)—and to focus on the distinct characteristics of SE of different students. We consider this an important issue, especially in an online and technologized environment in which students have access to myriad technological learning resources and English learning materials that articulate their SE beliefs.

### Influencing Factors of Students’ Self-Efficacy for Self-Regulated Learning in an Online Context

The study has revealed various factors that influence students’ SESRL in an online context, including task-, learner-, course-, and technology-level factors (RQ2). Among all the factors we identified, task-level factors seemed to be most closely related to the changes we observed in students’ SESRL. In Week 9, as the students became familiar with the online SRL process, accompanied by diversified English learning materials and tasks adjusted by the teacher, the majority were satisfied with the pace of online SRL and their performance in various tasks. This finding also corroborated the effect of students’ SESRL experiences in an online context observed in Britner and Pajares (2006), which indicates that the accumulation of positive experience in completing SRL tasks can help strengthen students’ confidence in online learning. In the last few weeks of our study, however, some students started to lose confidence in their self-regulation and language learning. A closer look at the tasks in the last few weeks revealed that the tasks designed were less varied and mostly concerned with English reading and writing activities, which might have been less attractive for the students.

In previous research, scant attention has been paid to the influence of tasks on students’ SESRL, particularly in the online environment. Our study emphasizes the important role of task-level factors (e.g., task design and task load) in affecting students’ confidence in self-regulated foreign language learning in an online context. According to our research findings, when students became familiar with the online SRL mode, the novelty effect was also likely to wear off (e.g., Ushioda, 2013), thus probably leading to their decreased SE for online SRL.

In addition to task-level factors, learner characteristics were also found to be critical factors influential for the students’ SESRL in the online course. In an online environment, students need to have the ability to autonomously engage in the learning process (Wang et al., 2013). In our study, self-discipline and self-adaptation emerged as two learner-level factors that exerted an effect on students’ SE for online SRL. Students with better self-discipline and self-adaptation strategies were found to have more confidence in concentrating on English learning, with or without distractions. Conversely, students’ SE for online SRL was negatively influenced by the lack of self-managing and self-monitoring skills, mirroring García Botero et al.’s (2019) finding that students’ motivation to study on Duolingo (a foreign language learning app) was disrupted by such shortcomings.

Furthermore, students’ SESRL was found to be contingent on course- and technology-level factors. For example, most students attached great importance to the online course and were quite positive in their acceptance of technology in the online SRL process, both of which were directly related to students’ greater confidence in overcoming distractions and technical issues. Our findings corroborated the positive relationship between technology-related SE and students’ learning reported in previous studies (e.g., Shen et al., 2013). The results further suggest that students’ willingness to accept the online learning platform could promote their SE beliefs for online learning in spite of technical obstacles.

### Pedagogical Implications

Our study’s findings can advance understanding of the dynamic nature of SE for online SRL in foreign language courses. The different levels of factors uncovered in the study also provide insights into the design and implementation of effective online SRL. First, EFL teachers should be fully engaged in providing support for students despite the fact they do not provide face-to-face instructions in the online SRL process. Mazzolini and Maddison (2003) noted that teachers’ involvement can both provide students confidence and promote their participation in online learning. For instance, teachers should observe students’ entire SRL processes on the online platform, and provide immediate support should the students lag behind or encounter technological problems.

Second, EFL teachers should be more attentive to students’ SE beliefs for online SRL, since the students are likely to show different levels of confidence in the online English learning tasks. As suggested by Joo et al. (2000), teachers can allocate instructional time and activities to strengthen students’ SRL strategies. For students who find it difficult to keep pace in online learning, teachers should offer them specific guidance in collecting and studying English learning materials, and in completing learning tasks more efficiently.

Third, various types of English learning activities should be designed based on students’ ability and pace of learning. González-lloret and Ortega (2014) suggest that teachers make full use of technology to develop pedagogic tasks that are difficult to implement in traditional classrooms. This can be realized by integrating multimedia for rich and authentic input and allowing students to produce their inputs in the target language in creative ways. In addition, appropriate assessment methods and timely feedback are necessary to encourage students to successfully complete their tasks and sustain their SE for online SRL (García Botero et al., 2019).

## Conclusion

This study offered a context-dependent, dynamic, and in-depth view of whether, how, and why EFL university students experienced changing SE beliefs in their online self-regulated learning process. However, our study has some limitations. The first limitation relates to its small sample size. A larger sample size with intensive longitudinal data collection in future research efforts may help advance our understanding of the dynamic nature of SE for online SRL. A related limitation concerns the course selected for this study, which was instructed by the same teacher. Future researchers may consider comparing online courses taught by different teachers to provide further insights into the role of the teacher in shaping students’ online SRL and their SE beliefs. Another limitation concerns the pedagogic tasks. Although we found a great influence of task-level factors on the students’ SE for online SRL, no further discussion was made about the effect of different task types. Future researchers could explore how different task designs may influence students’ SE and engagement in the online language learning activities.

In the field of foreign language learning, further understanding and exploration of students’ SE for online SRL and its antecedents (e.g., influencing factors) are needed to shed light on how learners’ SE can be improved for online SRL to achieve autonomous learning mindset in online contexts. Our work is particularly relevant in today’s increasingly technologized world where students have access to diverse and myriad online learning resources that resonate with their personal goals, learning interests and preferences, prior knowledge, and language and digital competence.

## Data Availability Statement

The data supporting the findings of the present study are available from the corresponding author upon reasonable request.

## Ethics Statement

All participants gave informed written consent prior to their participation in the study.

## Author Contributions

QX and JW contributed to the design of the study and writing of the manuscript. QX collected and analyzed the data. HP provided suggestions and modifed the manuscript. All authors contributed to manuscript revision and submission.

## Conflict of Interest

The authors declare that the research was conducted in the absence of any commercial or financial relationships that could be construed as a potential conflict of interest.

## Publisher’s Note

All claims expressed in this article are solely those of the authors and do not necessarily represent those of their affiliated organizations, or those of the publisher, the editors and the reviewers. Any product that may be evaluated in this article, or claim that may be made by its manufacturer, is not guaranteed or endorsed by the publisher.
